# Leveraging Green Ammonia for Resilient and Cost-Competitive Islanded Electricity Generation from Hybrid Solar Photovoltaic–Wind Farms: A Case Study in South Africa

**DOI:** 10.1021/acs.energyfuels.3c01950

**Published:** 2023-08-31

**Authors:** Victor N. Sagel, Kevin H. R. Rouwenhorst, Jimmy A. Faria

**Affiliations:** † Catalytic Processes & Materials, MESA+ Institute for Nanotechnology, University of Twente, Post Office Box 217, 7500 AE Enschede, Netherlands; ‡ Sustainable Process Technology Group, Faculty of Science and Technology, University of Twente, Post Office Box 217, 7500 AE Enschede, Netherlands; § Ammonia Energy Association, 77 Sands Street, Sixth Floor, Brooklyn, New York 11201, United States; ∥ Proton Ventures, Karel Doormanweg 5, 3115 JD Schiedam, Netherlands

## Abstract

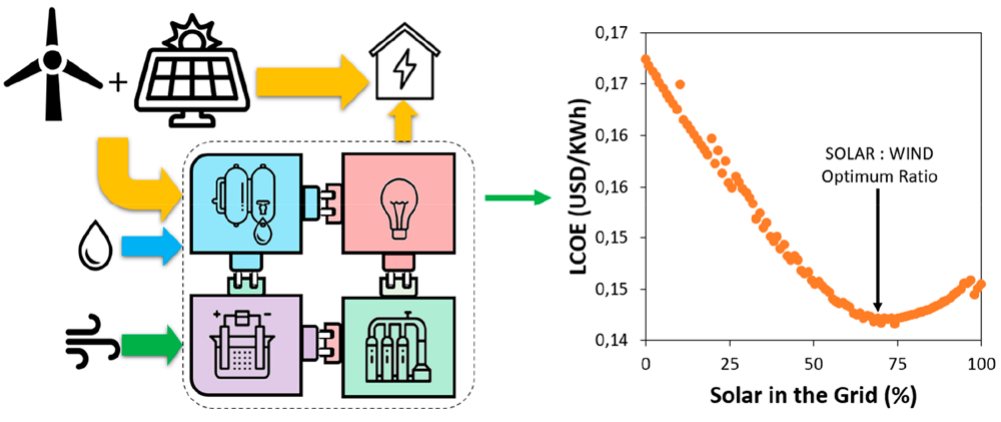

Hybrid solar photovoltaic (PV) and wind generation in combination with green ammonia as a seasonal energy storage vector offers an excellent opportunity to decrease the levelized cost of electricity (LCOE). In this work, an analysis is performed to find the most cost-effective configuration of power-to-ammonia-to-power (P2A2P). In P2A2P, wind and solar resources are combined with energy storage to design a resilient electricity grid. For daily generation, batteries are utilized for energy storage, whereas ammonia is employed to cope with seasonal fluctuations. The costs of energy storage capacity have a significant influence on the LCOE. Therefore, this work studies the effect of solar/wind hybrid generation systems and energy storage capacity on the LCOE. A base case of the region of De Aar in South Africa was selected because this inland location has excellent wind and solar resources. The optimized battolyzer and Haber–Bosch design capacity led to an overall load factor of 20–30%. At a 30% load factor, a hybrid system with 37% wind-based and 63% solar-based energy generation capacity was the most cost-effective configuration, resulting in a LCOE of 0.15 USD/kWh at a 5% annual discount rate. In an optimistic scenario for PV costs, the LCOE achieved is essentially unaltered (0.14 USD/kWh), while the contribution of wind and PV changes to 25 and 75%, respectively. This analysis indicates that appropriate designing of hybrid energy solutions will play a key role in determining the final energy storage capacities needed to reduce the LCOE. While these costs for LCOE are above those reported for coal-powered electricity in South Africa (e.g., 0.072 USD/kWh for businesses and 0.151 USD/kWh for households), a carbon tax of 50 USD/ton of CO_2_ can increase these costs to 0.102 and 0.191 USD/kWh, rendering a more promising outlook for the P2A2P concept.

## 1 Introduction

Human development is closely coupled to access of electricity. For this reason, it is not surprising that the electricity consumption per capita is one of the key indicators for health, productivity, safety, gender equality, quality of living, and education.^1^ In developing countries, increasing electricity generation and distribution capacity in the context of the energy transition brings a unique opportunity to deliver both clean power at a competitive cost and socioeconomic growth. In this context, Africa has an exceptional potential of solar (20 TW), hydro (350 GW), wind (110 GW), and geothermal (15 GW) energies.^1^ The International Renewable Energy Agency (IRENA) estimates that renewable-energy-installed capacity in Africa could reach 310 GW by 2030, avoiding the emission of ca. 310 million tons per year (MTY) of CO_2_ while increasing the electricity supply 2.5-fold.^2,3^ At the same time, ca. 600 million people in Africa have no access to electricity, representing 48% of the population on the continent. This is partly due to insufficient generation capacity and distribution networks.^2^ Despite the low CO_2_ emissions per capita in this region (0.8 ton of CO_2_/year), no other continent will be struck as severely by the impacts of climate change as Africa as a result of its geographical location, limited adaptive capacity, and poverty.^3^ Thus, for African countries, it is essential to make key investments in carbon-free generation capacity and power grids.

Mini-, micro-, and nano-electricity grids offer a unique opportunity to achieve this target in the African continent because installation and maintenance expenditures can be greatly reduced.^4^ To ensure resilient and on-demand electricity generation, these grids must include short- and long-term energy storage solutions. Batteries can mitigate the energy fluctuations between day and night when using photovoltaics (PVs) by facilitating short-term energy storage for daily as well as night-time operation and/or peak energy generation with high round trip efficiency (RTE) of 80%.^5^ However, the scalability of batteries to gigawatts is limited by cost (scaling factor of ∼1) and self-discharge issues that hinder their application for the required long-term energy storage in Africa. Carbon-free fuels (e-fuels), such as hydrogen (H_2_) and ammonia (NH_3_), on the other hand, are economically feasible in medium and large scales (scaling factor of ∼0.7), allowing months to a year of storage with RTE that varies between 20 and 40%. This is comparable to that of fossil-based fuels well-to-wheel RTE of 20%.^6^ The e-fuels have long suffered from the high cost of renewable electricity, reducing their economic potential to niche business models. Nevertheless, the impressive decrease in levelized cost of energy (LCOE) of PVs and wind, observed in the last years, has opened the door to e-fuels, particularly in places like the African continent, where the LCOE is predicted to be between 0.2 and 0.1 €/kWh for concentrated solar power (CSP), PVs, and wind.^7^

Hydrogen storage becomes more expensive than other e-fuels, like ammonia (NH_3_), after a single day primarily as a result of its volatility, flammability, and low density.^8^ Storing energy in the form of NH_3_ is promising and an often unavoidable step to reduce the final cost for implementing the highly fluctuating renewable electricity.^9^ Notably, NH_3_ is the only carbon-free vector that can be scaled-up in an economical manner from megawatt hour to terawatt hour using commercially available technologies.^10^ Furthermore, NH_3_ can be employed as both an energy carrier and fertilizer,^10−12^ which is particularly interesting for the African countries, where the utilization of N-based fertilizers is the lowest in the world.^13^ Besides, coupling renewable energy production with water purification technology can unleash the full potential of the agricultural sector of the African continent, which is arguably the largest employment sector in the region.^14^ Clearly, green ammonia has the potential to decarbonize the energy sector of Africa and enable low-cost and reliable access to fertilizers and fresh water for sustainable food production (i.e., energy–water–food nexus).^15^

The long history of industrial ammonia synthesis (+100 years) has led to well-established and safe protocols for production, storage, and transportation, facilitating its large-scale deployment as an energy carrier.^16^ The conventional Haber–Bosch (HB) ammonia synthesis process coupled to water electrolysis from green electricity can lead to an energy footprint as low as ca. 8 kWh/kg of NH_3_ at scales of ca. 105 kg of NH_3_/h. At the small scales (e.g., 0.5 kg of NH_3_/h), however, heat losses increase the energy consumption process up to 22 kWh/kg of NH_3_, which leads to down-scalability issues within the sustainability context.^17,18^ This is due to the high pressures (300–460 bar) required to achieve sufficient conversion at the elevated temperatures (400–550 °C) employed in the HB process.^18^ This issue is particularly relevant considering that decentralized electricity generation will play an essential role in decarbonizing the electricity mix in the African continent, where there are long distances between the power grid and remote communities.^4,19,20^

An attractive proposition to enable the down-scalability of the ammonia synthesis is the absorbent-enhanced Haber–Bosch (AE-HB) process. In this concept, the reactor is operated at moderate temperatures (370–400 °C) and lower pressures (10–30 bar).^21,22^ In contrast to the conventional process in which the ammonia-rich stream is separated using condensation after the reactor, in AE-HB, the small amounts of ammonia produced in the reactor are removed in an adsorption bed made of metal chlorides (e.g., CaCl_2_).^23^ Because the adsorption can take place at temperatures that are close to those used in the reactor, it is possible to reduce the energy penalty of ammonia separation. This reduces the energy consumption up to 50% at small scales and the investment costs as a result of the use of conventional steel for constructing the reactor. Recently, we showed that coupling this process with wind farms in the islands of Curaçao leads to cost-competitive and clean energy generation.^24^ We also showed that selecting the location for green ammonia production is key to achieve a feasible techno-economic solution. For instance, in locations with low wind energy resources, e.g., the island of Viti Levu in Fiji, it is more beneficial to import green ammonia from other locations to supplement local wind electricity production.^25^

Employing hybrid solar and wind solutions for the energy generation has shown great promise for reducing the LCOE in combination with green ammonia production using conventional HB in isolated regions in Argentina and Chile.^26^ Extrapolating these results to other regions is, however, not trivial, because the availability of electricity from wind and solar is strongly linked to the location of the plant. Furthermore, the power consumption profiles of each country are highly variable and often connected to heating and cooling demands, which are coupled to seasonal weather patterns.

In this context, the present contribution explores the interplay between the source of renewable energy (wind and solar PVs) and the extent of seasonal energy storage capacity. South Africa, one of the most developed countries in the continent,^27−29^ was selected as a showcase scenario because the majority of the power generation in the country relies on coal (>77%).^30^ The pollution caused by coal power plants results in nearly 2340 deaths per year in the country at a cost of 2.12 billion USD/year.^31^ For this reason, Eskom, the national electricity provider of the country and the largest in the African Union, has to decommission 10 of the 15 coal-fired power plants in the next 10 years.^32^ De Aar in South Africa [24.0° longitude (E) and −30.5° latitude (N)] was selected as a representative case study of a remote location in the African continent. This location has substantial wind and solar PV potential with a number of wind and solar PV plants under construction.^33^ Here, importing ammonia to the location is not feasible because this is a landlocked region with the closest ammonia factory at more than 600 km distance from Sasolburg. Thus, De Aar is assumed to be an island system without potential for ammonia import. Notably, our results revealed that curtailing of renewable energy generation is key to reduce the LCOE when using hybrid PVs and wind to generate 24–7 on-demand electricity with batteries and green ammonia for short- and long-term energy storage. This is primarily due to the large differences in generation profiles and costs of PVs and wind.

## 2 Methodology

**Figure 1:**
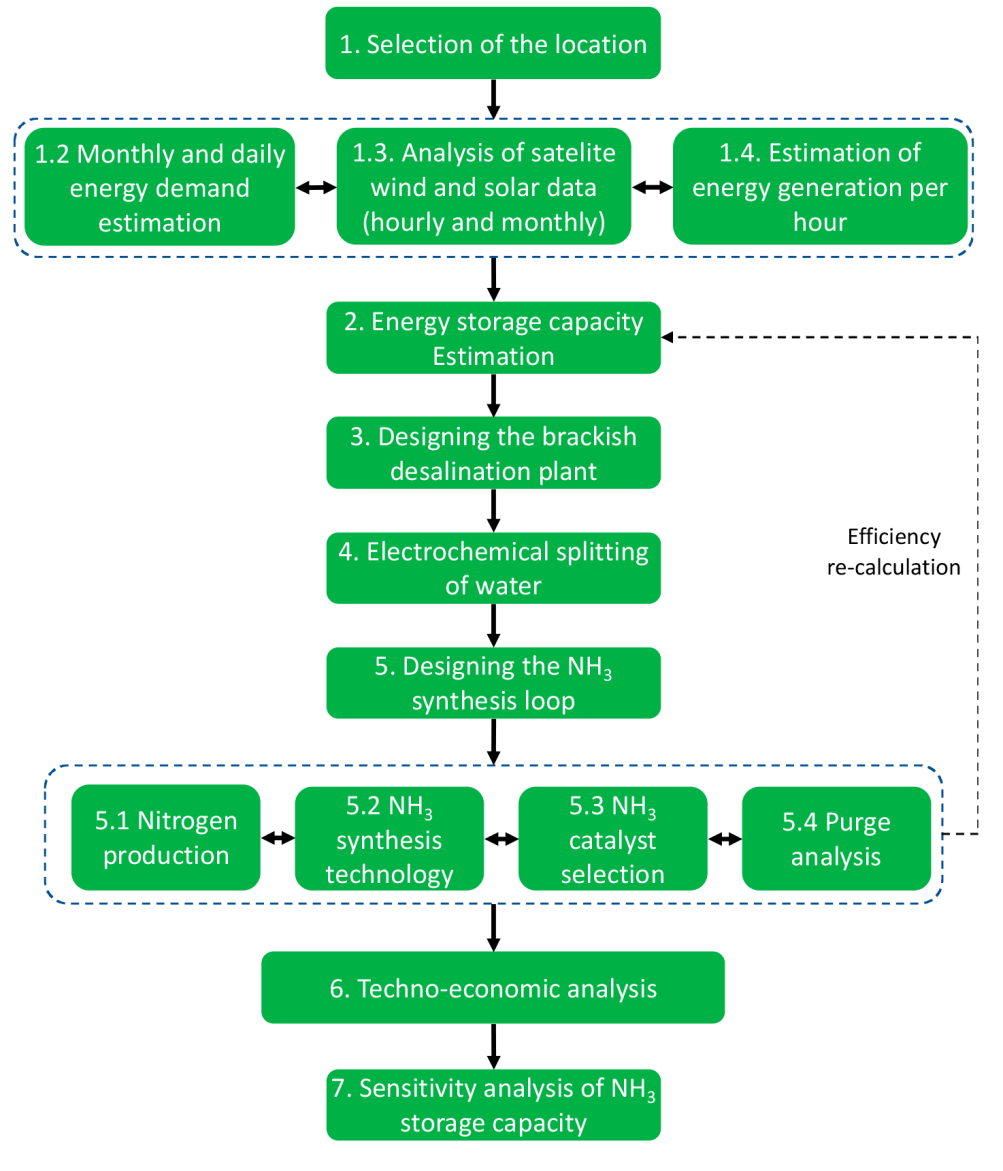
Designing and modeling approach employed for the determination of the P2A2P techno-economics for De Aar region in South Africa.

The dimensions and costs of the system have been quantified using an iterative algorithm previously developed in our group.^24,25^ The model has been extended to allow for electricity generation from solar PVs and wind (Figure 1). In this model, in step 1, a location for the energy system was selected (De Aar). For this location, energy demand data per hour have been retrieved and processed for daily and seasonal calculations. Wind and solar data have been retrieved from the Copernicus satellite,^34^ from which energy generation estimations are performed on the basis of solar/wind versus energy generation correlations. In steps 2, 3, 4, and 5, an order of magnitude estimation has been performed for the process equipment to make an initial design of the storage system to calculate its RTE. The design of the HB loop in step 5 was performed in Aspen Plus. On the basis of the RTE of the storage system, the demand data, and the wind/solar output data, an iterative model has been designed to recalculate the dimensions of the energy storage system and to recalculate its RTE. In step 6, a detailed techno-economic analysis has been performed to optimize the solar/wind hybrid grid system. At last, in step 7, a sensitivity analysis was performed to find the effect of equipment dimensions and costs on the optimal solar/wind ratio and its LCOE.

This model provides a novel route to design and optimize hybrid grid systems with reduced costs of dispatchable clean energy. The implementation of different types of renewable energy sources can result in positive synergies, in which a more stable and less cyclic generation pattern can be created. Furthermore, energy storage is a major cost factor for reliable renewable energy systems.^35^ Here, optimization between generation capacity and storage capacity could reduce the overall costs of reliable energy. The creation of a mode that optimizes both effects could lead to insightful considerations for new renewable energy grid designs.

In this model, batteries are used for energy storage on the hourly range and ammonia is used for energy storage on the seasonal scale.^36^ For short-term storage, battolyzers are selected because these systems can act as electrolyzers and batteries with high energy efficiency. Battolyzers, at first, store excess energy in Ni–Fe batteries and subsequently turn into electrolyzers once the batteries are fully charged.^36^ The power-to-ammonia-to-power (P2A2P) model is based on a process design for power conversion into ammonia (power-to-ammonia) using an AE-HB process combined with battolyzers, and subsequently, an ammonia-to-power system based on solid oxide fuel cells is employed to generate electricity in the end of the cycle.^37,38^ AE-HB is selected over the conventional HB process as a result of its flexibility, which allows for intermittent production of ammonia and its lower operation and capital costs at medium and small scales.^6,24,39^

The ERA5 Reanalysis database from the European Union (EU) project Copernicus^34^ has been used for the weather pattern data from South Africa, with 3 years of data used (2019–2021). Data from the South African utility company Eskom Holdings SOC has been used for the demand profiles.^40^ The demand curves for 2021 are used in this case. Eskom has a market share of 95% in the South African electricity market; thus, these data provide a representative picture of the electricity consumption in the country.^41^ In this case, it has been assumed that the P2A2P must supply 1% of the contracted demand following the national demand pattern published by Eskom. This provides a realistic scale for the local grid of the region of De Aar in South Africa. For wind to energy output correlations, the Vestas V82 1.65 MW wind turbine is modeled as a representative and conservative system of inland wind farms.^42^ A linear correlation between solar radiation and PV output at 15% efficiency is assumed for the PV generation capacity.^43^ Additional information regarding the mathematical description of the model and calculation methodology can be found in sections 1 and 2 of the Supporting Information.

The LCOE has been determined using the capital expenditures (CapEx), operational expenditure (OpEx), and annual discount factor of 5%. For the CapEx and OpEx, standard cost correlations from the book “Plant Design and Economics for Chemical Engineers” have been utilized.^44^ Furthermore, for the CapEx, a Lang factor has been implemented to correlate bare equipment costs (BEC) to total CapEx costs.^45^ A Lang factor of 4.105 has been utilized on the basis of previous work by our group.^24^ For the solar and wind farm costs, the installed costs have been estimated using International Energy Association (IEA) estimates for South Africa or economic assessments from other companies. For wind turbines, an installed cost of 1877 USD/kW has been estimated.^46^ For solar energy, installed costs of 961 and 618 USD/kW for base case and good case scenarios have been estimated, respectively.^47,48^ The effect of solar CapEx on LCOE has been illustrated in the Supporting Information. Further readings into the utilized cost estimation method including bare erected cost (BEC) estimation methods can be found in the Supporting Information of the previous work from our group.^24^

**Figure 2:**
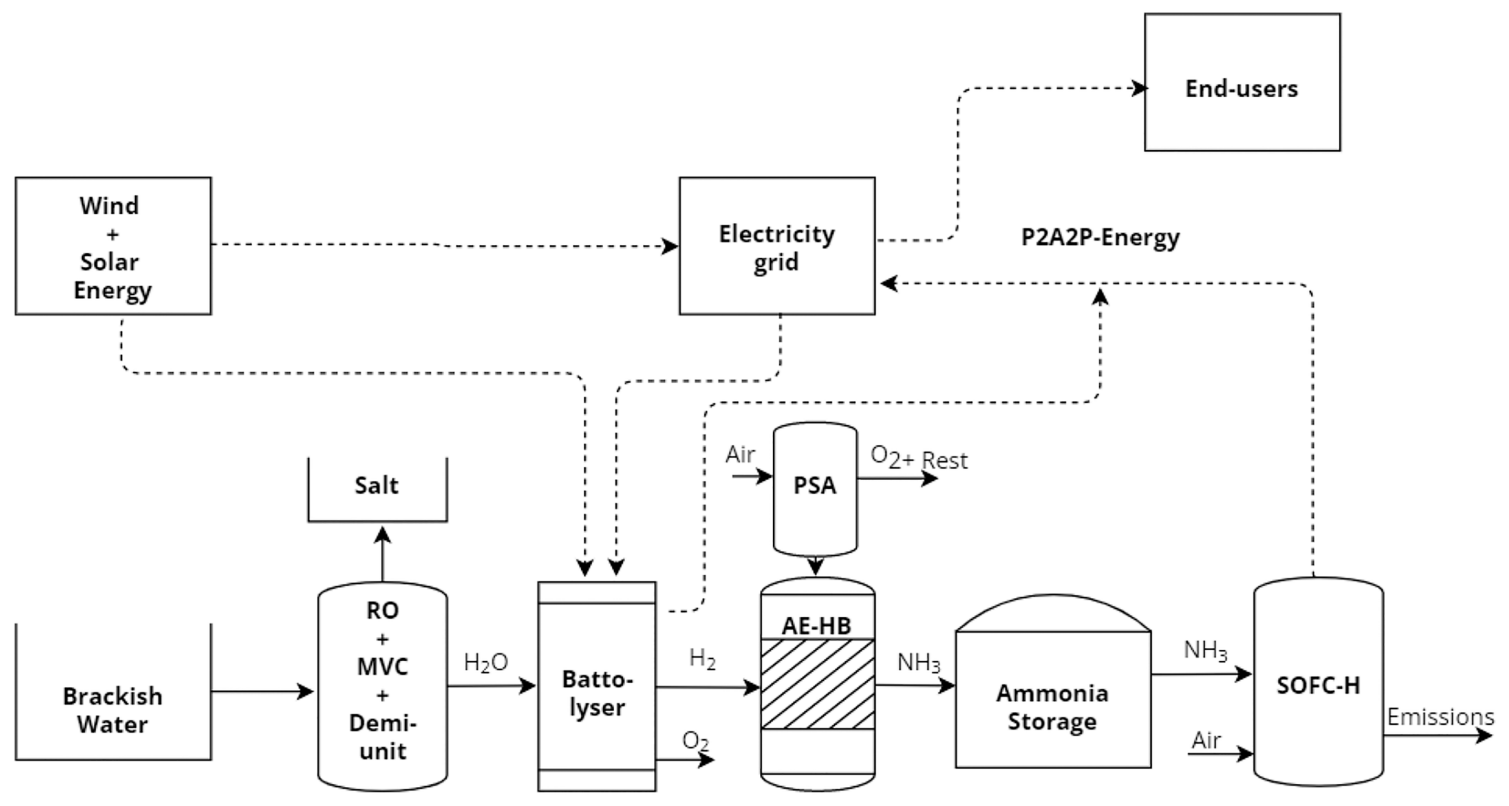
Conceptual process flow diagram for P2A2P, using a battolyzer, pressure swing adsorption (PSA), and AE-HB. This figure was reproduced with permission from ref (24). Copyright 2023 Elsevier, Ltd.

From the combination of the round-trip efficiency (RTE) of the battery storage system, the P2A2P storage, the wind and solar patterns, and the electricity demand curves, it was possible to optimize the system for reliable electricity supply throughout the year solely based on renewable power input. An overview of the system is shown in Figure 2. A more detailed description of the technologies chosen for the P2A2P system can be found in section 1 of the Supporting Information.

Dependent upon the current electricity generation and demand from renewables, a certain amount of excess energy can be utilized to produce ammonia for long-term energy storage. Because the RTE of ammonia energy storage is ca. 24% compared to 85% for battolyzers, the battolyzer can reduce the LCOE by minimizing the fraction of energy required for long-term storage, i.e., reducing the size of the electrolyzers and ammonia synthesis sections. For this reason, an energy storage system designed to achieve the maximum charging rate will have a large CapEx and a low average utilization factor. Consequently, this system will have a high LCOE. In this scenario, the low prices of wind and solar PVs have opened new possibilities to lower the cost of green electricity. Therefore, it could be economically beneficial to strategically undersize energy storage equipment and to oversize the amount of wind and solar generation capacity, with some curtailment to compensate for the energy losses.

## 3 Results

**Figure 3:**
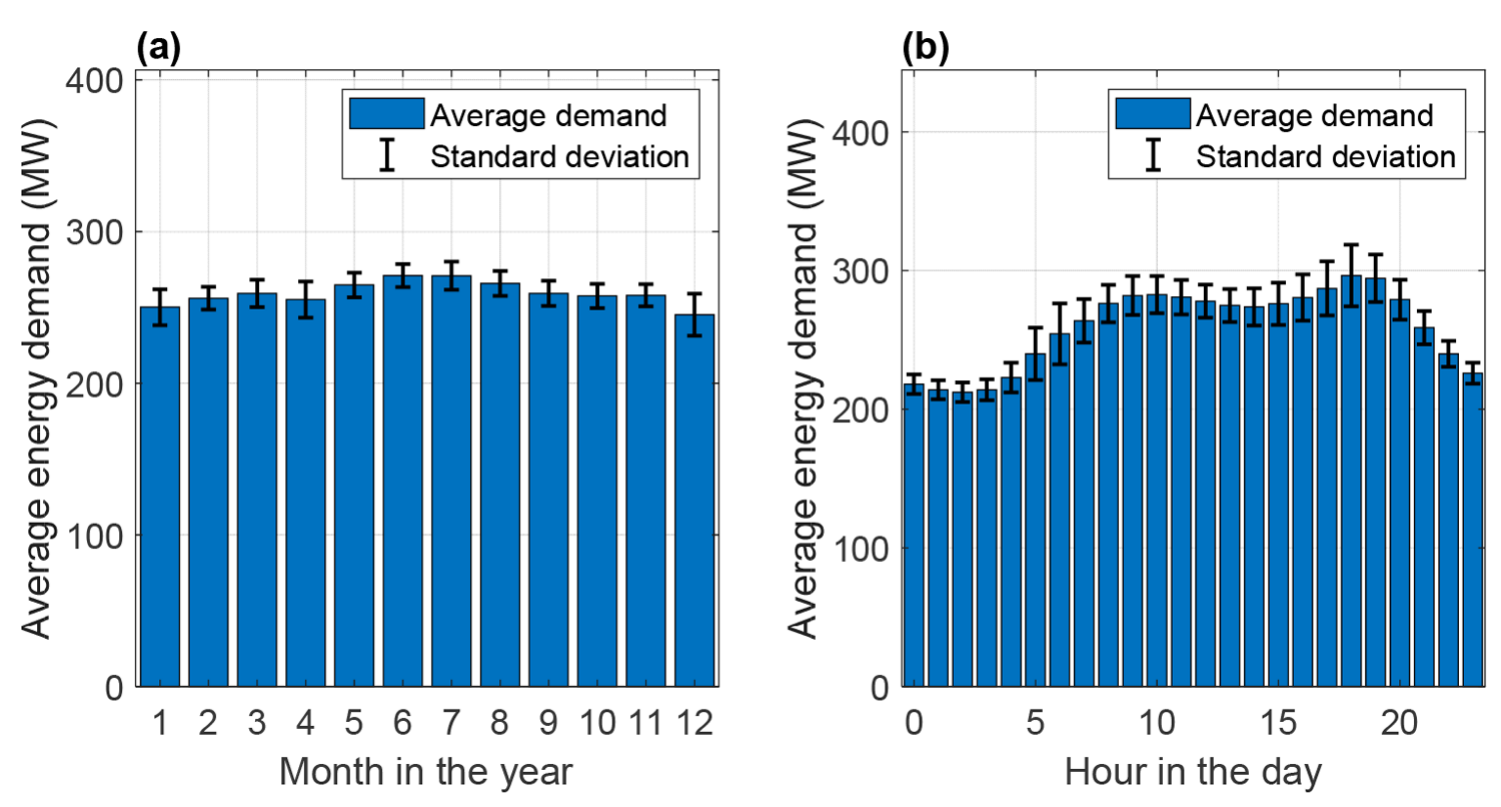
Average energy demand per (a) month and (b) hour in South Africa scaled to 1% of the total demand data reported by Eskom in 2021.

The hourly and seasonal demand patterns, scaled down to 1% of the total values, were generated from the data published by Eskom (see Figure 3). As noted, the energy demand varies from ca. 245 MW in the summer period to 271 MW in the winter season. This increment is associated with the higher energy consumption in the cold months of the winter in the southern hemisphere (i.e., from June to August). As expected, on an hourly scale, there is a peak demand in the mornings and evenings. For instance, at 3:00 a.m., the demand is as low as 212 MW, whereas at 6:00 p.m., the average demand peaks at 296 MW.

**Figure 4:**
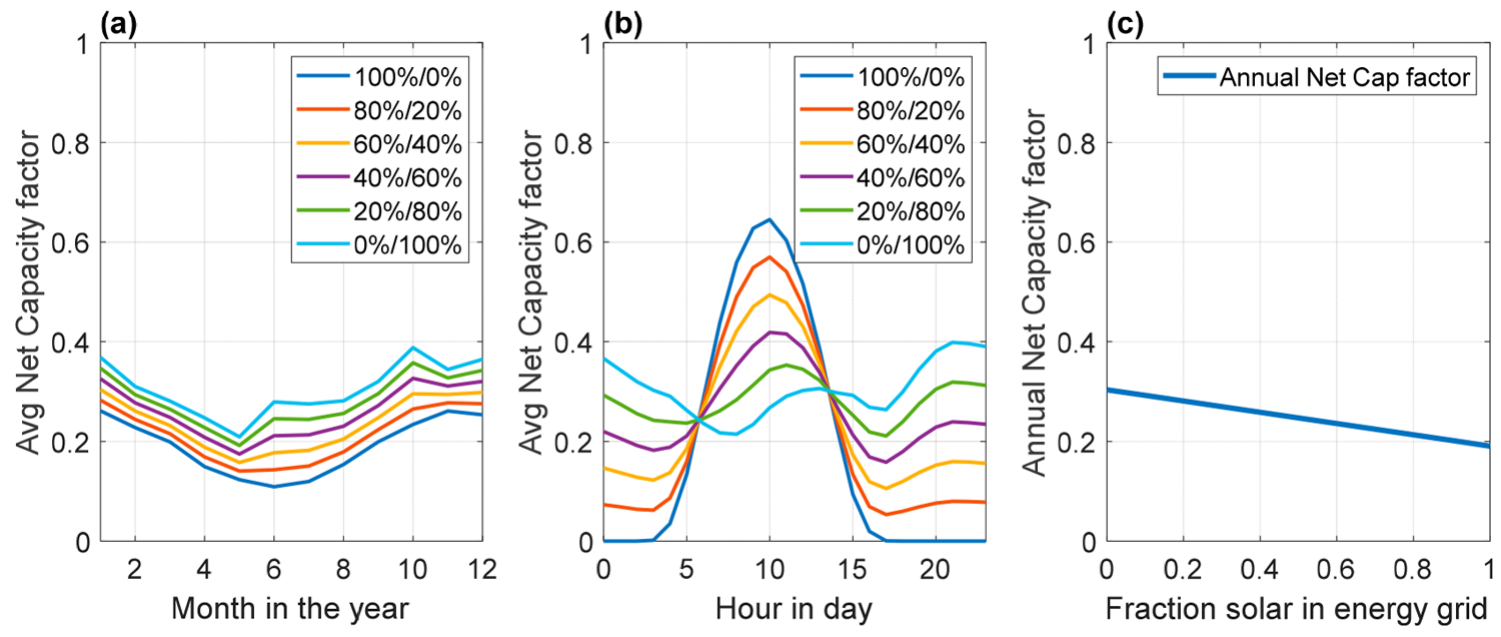
Average net capacity factor of solar/wind hybrid systems on a (a) seasonal scale and (b) hourly scale, together with the effect on the (c) annual net capacity factor.

With the combination of the ERA5 data on average hourly solar radiation and wind speed, one can estimate the capacity factor of solar PV and wind energy generation, respectively. Figure 4 illustrates the net capacity factor of solar and wind hybrid energy on hourly and seasonal scales, which are based on the wind to energy output pattern of the Vestas V82 1.65 MW wind turbine as well as the linear solar radiation to solar energy generation correlation (see sections 1 and 2 of the Supporting Information). The net capacity factor is defined as the average generation output divided by the maximum generation output for a certain moment in time. Surprisingly, solar PVs and wind are partly complementary on the daily basis as it can be observed in Figure 4a, where the lowest net capacity factor for the 0% solar/100% wind case coincides with the peak on solar PVs in the 100% solar/0% wind case. The results suggest that on a daily basis, 80% solar with 20% wind leads to the least cyclic average output. On a monthly basis, this complementarity diminishes, because both solar PVs and wind have the minimum power output in the middle of the year, when the demand for power is at its maximum (Figure 4b). This asynchronous availability of renewable energy and average monthly peak demand can only be managed using seasonal energy storage.

**Figure 5:**
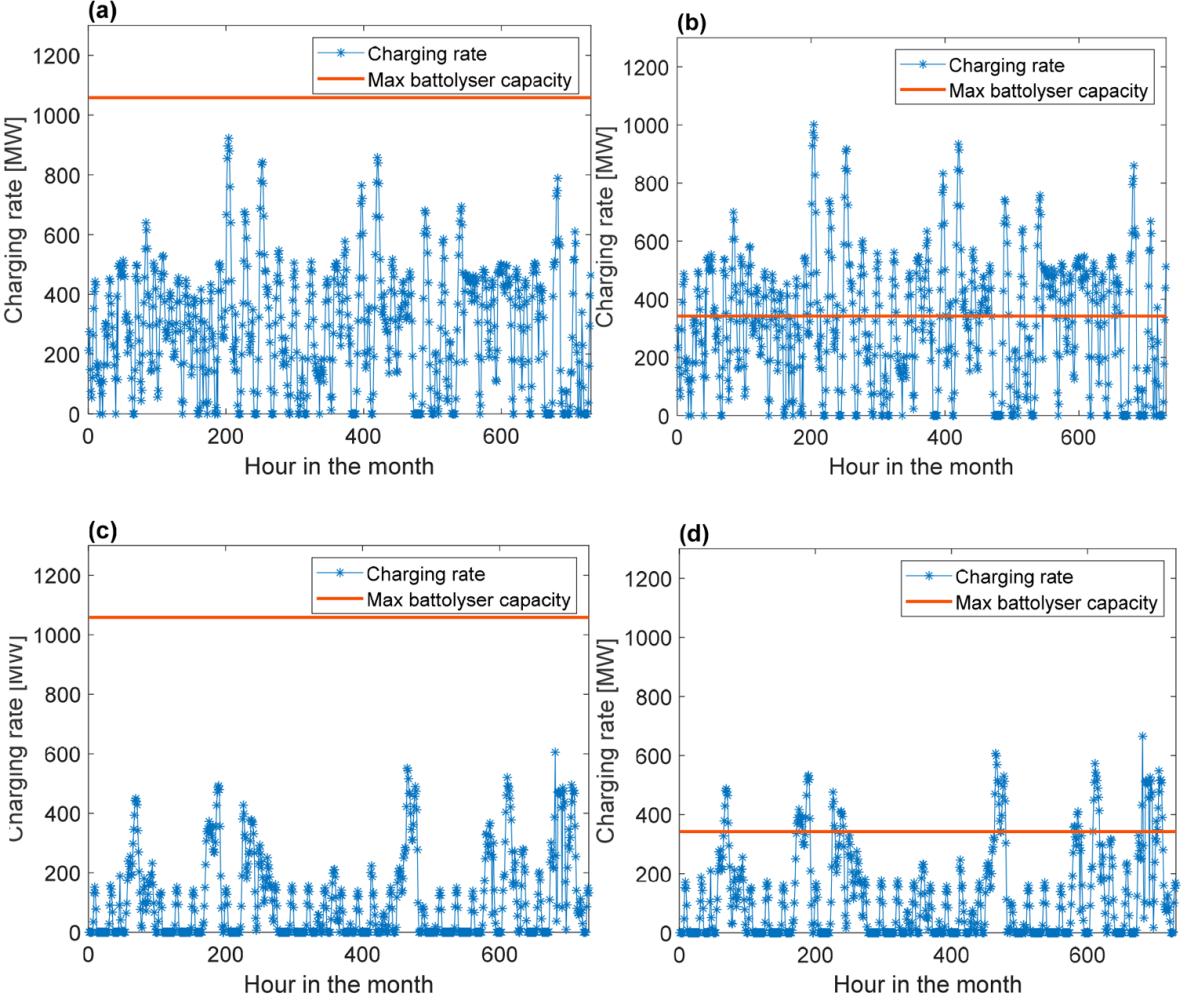
Charging rate data for a 50% wind/50% solar hybrid design using real data for January 2021 with the (a) maximum charging rate at full design capacity together with the real charging rates and (b) maximum charging rate at 30% charging capacity compared to full design capacity together with real charging rates. (c and d) Panels a and b, respectively, for June, per reference.

Using the given data for demand, wind energy output, and solar energy output, it is possible to design a P2A2P system. It is not economically attractive for the short- and long-term charging mechanism to be designed at full capacity. Full capacity is defined as the charging rate at 100% net capacity in solar and wind energy generation equipment, at the lowest feasibly possible grid demand. Panels a and b of Figure 5 illustrate the real charging capacity and the maximum charging capacity as a visualization of how often a given charging capacity is reached. It is important to mention, however, for the purpose of readability, that only a single month is illustrated (January). Because this month falls in the southern summer season, relatively high-capacity factors are obtained. For reference, the results for June are illustrated in panels c and d of Figure 5.

A case with a smaller system, set at 30% of the base case, is also shown in Figure 5. In this case, part of the electricity generated by solar PVs and wind is not stored, resulting in curtailment of electricity generation. Essentially, in this case, the solar PV and wind generation plants are purposely oversized. The underlying motivation here is to explore the impact of curtailment on the LCOE because this can potentially help to increase the utilization factors of expensive batteries, electrolyzers, ammonia synthesis loop, and ammonia to power conversion systems.

**Figure 6:**
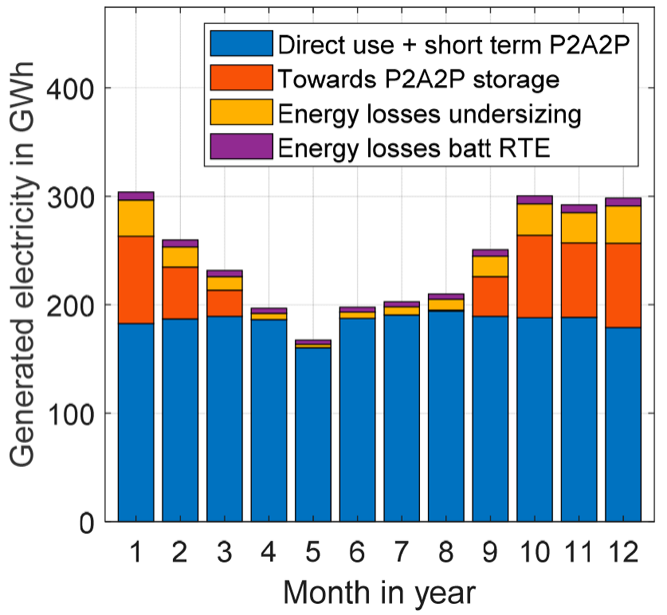
Monthly energy generation patterns and end use of the given energy generation for a 50% wind and 50% solar system at 30% battery and HB capacity compared to the full-scale design.

Next, the system was recalculated using an iterative algorithm that finds new optimum sizes for wind, solar, and storage capacities. This was performed to compensate the energy losses resulting from the undersizing of the energy storage equipment (see section 2 of the Supporting Information). As an example, the generated electricity and the energy storage capacity requirement for the 50% wind and 50% solar PV case at 30% charging capacity are shown in Figure 6. Here, it can be observed that a large excess of energy should be generated to compensate for the energy shortages from April to July. For every month, the losses in the battolyzers are first subtracted from the generated amount of energy within a certain day. Subsequently, for a day in which an excess of generated energy is found, after charging sufficiently for the predicted energy deficit during the night and parts of the evening and morning, the excess energy is flown toward ammonia generation. During periods with energy shortages, battolyzers cope with intraday fluctuations, whereas the solid oxide fuel cells deliver a more stable baseload of energy from stored ammonia to compensate for the lower current energy generation.

**Figure 7:**
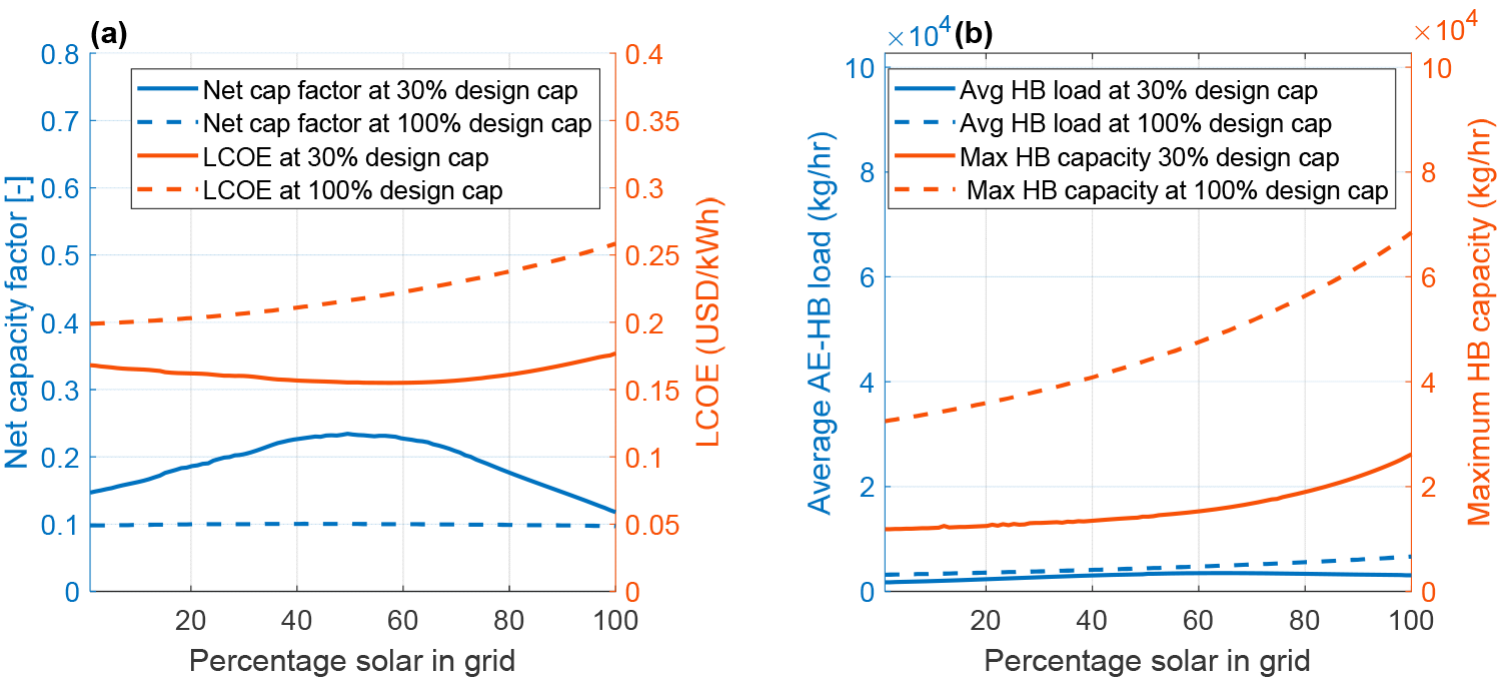
(a) Net capacity factor and LCOE at 30 and 100% battolyzer + HB plant capacity together with (b) average HB load and maximum HB capacity at 30 and 100% battolyzer + HB plant capacity.

The LCOE and net capacity factor of the battolyzer and AE-HB process can be estimated for each one of the possible combinations of solar and wind electricity generation at 30 and 100% of the peak capacity of the hybrid electricity generation plant (see Figure 7). Here, it was observed that, when the system is designed to utilize 100% of the peak capacity of renewable energy, the net capacity factor of the battolyzer and AE-HB is essentially independent of the fraction of solar PVs in the grid. Surprisingly, a maximum in net capacity factor was observed when curtailing renewable energy was considered, i.e., when the hybrid generation plant is designed for only 30% of the maximum generation capacity. This can be explained by the difference in the required average AE-HB load and maximum load capacity obtained for each scenario. At the same time, in Figure 7a, one can note that the lowest LCOE is achieved at 63% solar in the grid at 30% design capacity, whereas a full wind farm grid is the cheapest option at 100% design capacity. The latter option, however, results is higher LCOE. This analysis shows that it is possible to reduce the energy generation costs using inexpensive solar PVs in combination with wind and increase the utilization factor of the battolyzers and AE-HB system, which is dependent upon the design capacity of the power generation section.

To identify the underlying cause of the differences in the LCOE for the 100 and 30% design capacity scenarios, one could estimate the average load of the AE-HB and the maximum AE-HB capacity as a function of the fraction of solar PVs in the system (see Figure 7b). Here, one can note that the correlation with the maximum AE-HB capacity is exponential for the system with 100% design capacity. This is due to (1) the marked cyclic generation output pattern of solar PVs that results in large generation peaks and, therefore, greater installed capacity of battolyzers and AE-HB units, (2) larger short-term energy losses in the process that must be compensated for with additional energy generation equipment and, thus, greater battolyzer and AE-HB capacity as the solar fraction increases. At 30% design capacity, however, the maximum AE-HB capacity values increase faster at higher levels (>60%) of solar in the grid. This is due to the undersizing of the battolyzer + HB capacity, leading to larger fractions of energy curtailment at higher solar levels. This results in a stronger correlation between additional solar in the grid and additional energy losses as a result of undersizing and, therefore, a stronger correlation with additionally required energy generation equipment to compensate for energy losses. Essentially, the coupling between scaling of the energy generation installation, energy curtailing, and energy losses as a result of the short-term and seasonal storage dictates the maximum plant capacity of the hydrogen generation and ammonia synthesis sections.

For the average level of the ammonia load, a different trend can be found. At low levels of solar in the grid, while adding more solar capacity, larger energy shortages will be present in the winter months as a result of the more cyclic behavior of solar energy compared to wind energy, and thus, a larger overall ammonia production is required to compensate for this change. Consequently, greater energy generation capacity is required to account for this increase in ammonia demand, which, in turn, generates more energy during the winter months and, thus, reduces the ammonia demand. At higher levels of solar, however, the ammonia demand will either reduce or increase to a lesser extent depending upon the design capacity. Furthermore, an optimum between the cost of energy storage and the cost of energy generation was found at a design capacity of 30%. At this design capacity, a system with 37% wind and 63% solar results in the lowest possible LCOE of 0.15 USD/kWh (see section 4 of the Supporting Information). For the given optimal configuration, an additional sensitivity analysis is performed to illustrate the effect of equipment costs of the major constant contributors on the final LCOE (section 4 of the Supporting Information).

## 4 Discussion

Broadly speaking, these results suggest that, in the base case scenario for solar PV costs and optimistic scenario, a hybrid system results in the lowest LCOE. For the base case scenario, a 37% wind and 63% solar PV-based energy generation capacity system results in the lowest LCOE (∼0.15 USD/kWh). For the optimistic case scenario, a 25% wind and 75% solar-based energy generation capacity system results in a slightly lower LCOE (∼0.14 USD/kWh). Notably, this indicates that the reduction in the costs of the PV system from 961 USD/kW^47^ in the conservative scenario to 618 USD/kWh^48^ in the optimistic has nearly no impact in the final LCOE. For both cases, a battolyzer + AE-HB design capacity of 20–30% of the theoretically assumed realistic maximum is found to be the optimum between battolyzer + AE-HB CapEx and wind/solar PV-based energy generation CapEx. This is a compromise between the added CapEx for renewable electricity generation capacity and utilization of the peak capacity for the energy storage system. Despite the lower LCOE of the hybrid system, it is possible that a monosystem could become the most profitable option if one of the two power generation methods becomes significantly cheaper in the future. At the same time, hybridization offers additional benefits in terms of resilience to the grid, which is difficult to achieve with monosystems.

In this work, a location within South Africa was taken as a base case. As illustrated in Figures 2 and 3, seasonal demand is relatively consistent and solar output is significantly lower in the winter months of the southern hemisphere. It is hypothesized that energy demand is relatively consistent throughout the year because South Africa does not become very cold in winter. It is expected that solar will become less efficient in (colder) countries that have a strong seasonal energy demand and strong anticyclic solar-based energy generation output, resulting in grid hybrids with relatively more wind-based energy present.

As stated before, a LCOE of 0.14–0.15 USD/kWh can be achieved with the proposed system. This illustrates that P2A2P is an interesting concept for islanded communities within South Africa that do not have a grid connection to obtain reliable electricity. The electricity price in South Africa is about 0.072 USD/kWh for businesses and 0.151 USD/kWh for households as of 2023.^49^ A potential carbon tax could make P2A2P more economically competitive. For example, a carbon tax of 50 USD/ton will add 0.04 USD/kWh to the cost of coal-based energy.^25^ Apart from the economic incentive, P2A2P also has a significant benefit over coal-based energy in South Africa, because fine dust results in 25 800 (19 700–30 000) deaths per year in South Africa.^50^

To reduce the LCOE of P2A2P further, it will be essential to focus on the following issues: (1) Increasing the efficiency of electrolyzers and solid oxide fuel cell (SOFC)–H fuel cells should be prioritized because the efficiency of energy storage in ammonia is ca. 22%_LHV_ as a result of the losses in these two units. (2) Further reductions in the costs of battolyzers and SOFC–H fuel cells are necessary to increase profitability. (3) A decrease in the installed costs of wind- and solar-based energy generation equipment facilitates technological uptake in developing countries. Next to the economic challenges and opportunities of green ammonia as an energy storage vector, one must also consider the potential environmental and climate change advantages of this technology. Here, one can anticipate that the implementation of green-ammonia-based technologies can lead to CO_2_ reductions of 96–99% compared to coal- and heavy-fuel-fired power plants that have large carbon footprints (0.78 and 0.92 kg of CO_2_ equiv/kWh for heavy fuel and coal, respectively).^51,52^ Therefore, for countries like South Africa with a historic reliance on coal, it will be key to adopt aggressive initiatives to deploy green power generation. More broadly, in sub-Saharan countries, the potential for carbon avoidance using P2A2P technology is massive because the installed power capacity in this region is expected to grow by 240 GW in the next 2 decades.^53^ To fuel this endeavor, it will be key to securing substantial financial support from developed economies. Unfortunately, matching the level of financial assistance required to achieve the energy transition targets has been an elusive issue.^54^ For this reason, we anticipate that the lower financial thresholds required for developing decentralized power generation, such as the one proposed herein, can facilitate the implementation of green energy generation in the developing world.

## 5 Conclusion

Hybrid solar PV and wind generation has shown to be an excellent opportunity to decrease the LCOE, which, in turn, can lower the production costs of green ammonia as a seasonal energy storage vector for decentralized electricity generation. In this work, an analysis was performed to find the most cost-effective configuration of P2A2P. In P2A2P, wind and solar sources are combined with energy storage to design a resilient islanded electricity grid. This work has focused on finding an economic optimum between the costs for storage equipment and those for energy generation equipment as well as the optimum of wind and solar-based energy fractions in a hybrid system. An optimized design consisting of battolyzers and AE-HB was found to have an overall load factor of 20–30%. A hybrid system with 37% wind and 63% solar-based energy generation capacity results in the most cost-effective configuration with a LCOE of 0.15 USD/kWh at a 5% annual discount rate when using a 30% load factor for the renewable energy generation plant. In an optimistic scenario for PV costs, a LCOE of 0.14 USD/kWh can be achieved using a hybrid system with 25% wind and 75% solar-based energy generation capacity. Furthermore, P2A2P provides an excellent solution to reduce the environmental pollution and CO_2_ emissions of existing coal-fired power plants in South Africa.

## Supporting Information Available

General methodology, process equipment selection, energy efficiency estimations, solar and wind patterns, and sensitivity analysis (PDF)
