# Supplementary material for: Leveraging Green Ammonia for Resilient and Cost-Competitive Islanded Electricity Generation from Hybrid Solar Photovoltaic–Wind Farms: A Case Study in South Africa
Source: Energy Fuels. 2023 Aug 31;37(18):14383–92. doi: 10.1021/acs.energyfuels.3c01950 (PMC10518817; doi:10.1021/acs.energyfuels.3c01950)
Supplement: Supplementary file 1 — ef3c01950_si_001.pdf [file ef3c01950_si_001.pdf]

Leveraging Green Ammonia for Resilient and Cost Competitive Islanded Electricity Generation from Hybrid Solar PV-Wind Farms: A study case in South Africa

SUPPLEMENTARY ONLINE MATERIAL

Victor N. Sagel[1,2*], Kevin H. R. Rouwenhorst[1,3,4*], Jimmy A. Faria[1*]

Affiliations:

[1] Catalytic processes & Materials, MESA+ Institute for Nanotechnology, University of Twente, P.O. Box 217, 7500 AE Enschede, The Netherlands

Sustainable Process Technology group, Faculty of Science and Technology, University of Twente, PO Box 217, 7500

AE, Enschede, The Netherland

[2]Sustainable Process Technology group, Faculty of Science and Technology, University of Twente, PO Box 217, 7500 AE, Enschede, The Netherlands

[3]Ammonia Energy Association, 77 Sands Street, 6th Floor, Brooklyn, NY 11201, USA

[4]Proton Ventures, Karel Doormanweg 5, 3115 JD Schiedam, The Netherlands

*** Corresponding authors:**

Victor N. Sagel: v.n.sagel@utwente.nl

Kevin H. R. Rouwenhorst: k.h.r.rouwenhorst@utwente.nl

Jimmy A. Faria: j.a.fariaalbanese@utwente.nl

Index

1. General methodology: 3
2. Energy efficiency estimation 4
3. Solar and wind patterns 5
4. Sensitivity analysis 6
5. References 8

1. General methodology:

Figure 1 illustrates a process scheme that starts with the demineralization of brackish water to subsequently utilize for electrolysis of water. For the demineralization, a Zero-Liquid-Discharge (ZLD) method is utilized as proposed in [1]. The ZLD process combines High Rejection Reverse Osmosis (HRRO), together with Low Rejection Reverse Osmosis (LRRO) as described in [2], and Mechanical Vapor Compression (MVC). In the next step, battolysers are utilized to store energy on a short term scale, and to produce hydrogen. The hydrogen gas is subsequently utilized to thermal-catalytically produce ammonia in the AE-HB process. The nitrogen for ammonia production is obtained using Pressure Swing Adsorption (PSA), due to its high flexibility and reasonable energy consumption [53][3]. For the ammonia storage facility, ammonia is stored in large scale refrigerated storage tanks at atmospheric pressure and -33 ^{o}C. For large-scale ammonia storage, refrigerated storage at atmospheric pressure is the most cost effective and common storage method, alternatives are high pressure storage at room temperature and storage is salts [4].

To convert ammonia back into electricity, several process options are possible. The two main options are utilizing direct fuel cells, or fuel cells with thermal energy recovery. From the given options, utilizing direct ammonia fuel cells utilizing Solid Oxide Fuel Cells are the preferred option[5]. Heat recovery technologies are not preferred due to their poor electrical efficiency gain, together with significant increase in CapEx [6]. The efficiency of the SOFC is assumed to be 55%$_{LHV}$ in this work [7]. The utilization of direct ammonia fuel cells is possible as the electrocatalyst in SOFC is Ni-based and does not require pre-dissociation as Ni-based electrolysis is able to dissociate NH_3 at temperatures present within SOFC [8]. This makes direct fuel cell technologies attractive, as external ammonia cracking can result in heat and thus efficiency losses[7].

The specific energy consumption (SEC) of ammonia production was 12.0 kWh/kg NH_3, as calculated in [9] (see **table 1**). Modern ammonia plants based upon natural gas or coal have an energy consumption of 7.8 and 10.6 kWh/kg NH_3, respectively. Utilizing SOFC-H with an electrical efficiency of 55%$_{LHV}$, an RTE of 24% is found for seasonal storage. Although this is deemed low, it is higher than the well-to-power (W2W) efficiency of fossil based technologies, which often range in 16-20% [10].

Table 1: Energy consumption of the P2A2P process, in kWh/kg NH_3, from [9].

Process	Energy in kWh/kg NH_3	Reference
Water desalination	0.02	[11,12]
Electrolysis	8.75	[13]
PSA	0.35	[14]
Ammonia synthesis loop	2.76	Adsorption beds [15], rest Aspen Plus
Ammonia storage	0.16	[16,17]
**Total**	**12.0**	

2. Energy efficiency estimation

For a full capacity system, the model is designed so that sufficient electricity is charged to cope with daily energy cycli, assuming an averaged cyclus of generation and demand, and a battolysers RTE of 85%. With reducing battolyser capacity, the required amount of short term energy storage will reduce as well. Because of this, ammonia is required to compensate for short term energy shortages. The amount of short-term ammonia losses as a fraction of the total charging rate is calculated for each hour in 2019 to 2021 according to:

$$E(n)_{loss,fraction} = \max\left(\left(\frac{E(n)_{charge,hourly} - E(n)_{maxcharge,hourly}}{E(n)_{charge,hourly}}\right), 0\right)$$

With $E(n)_{charge,hourly}$ defined as the difference in energy generation and energy demand in a given hour of the year.

Subsequently, the amount of energy that is lost in a year, and should be compensated using ammonia is calculated according to the following equation:

$$E_{ammonia} = \sum_{n=1}^{8760} E(n)_{loss,fraction} * E(n)_{charge,hourly}$$

The average of the three years is taken as the additional amount of energy that is required to be generated by ammonia. On the seasonal scale, a similar calculation is performed to find

how electrolysis and Haber-Bosch under sizing effects ammonia production. With the two loss factors combined, one can calculate the increase in energy generation capacity required to calculate the ammonia quantity, and ammonia generation capacity required to design an islanded electricity system.

For the weather data, data from 2019, 2020, and 2021 has been utilized from the Copernicus database with 1 data point per hour, resulting in a string of. Specifically, the solar radiation (total sky direct solar radiation at surface) and the wind speed (U- and V-direction wind speed at 100 m hub height) have been retried. This results in a total of 26.304 x 3 = 78.912 data points that are used as an input in this work. For the demand pattern, data is obtained from the previous 5 years, up to the middle of 2022. Due to the non-complete data from 2022, this year is disregarded. Furthermore, the demand data from 2020 is disrupted due to the COVID lockdown, and the demand from before the lockdown is deemed to be outdated. Therefore, data from the complete 2021 year is taken as an input for demand patterns. For wind to energy output correlations, the Vestas V82 1.65 MW wind turbine [18] is modelled, whereas a linear correlation between solar radiation and PV output at a 15% efficiency is assumed for the PV generation capacity [19]. For wind and solar CapEx estimated, data from [20] and [21] are utilized, respectively. Cost estimates for PV vary widely in literature, therefore a good case scenario from [22] is utilized in this work as well. For OpEx, the wind turbine OpEx costs from [23] are scaled linearly with the CapEx from the South Africa case, as compared to the CapEx from the given source. For PV OpEx, a constant 1.1% annually from the CapEx is used [22].

3. Solar and wind patterns

From the Copernicus database, solar and wind data has been retrieved from 2019, 2020, and 2021. The data has been processed and are visualized in **figure S1**. From **figure S1**, it shows that solar energy is highly cyclic, on an hourly scale as well as on a seasonal scale. Wind energy, on the contrary, is more stable throughout a day and the year. On a daily basis, solar PV and wind are complementary, noting the relatively low wind speeds during the day, when solar PV has a peak capacity. On a monthly basis, solar PV and wind are less complementary.

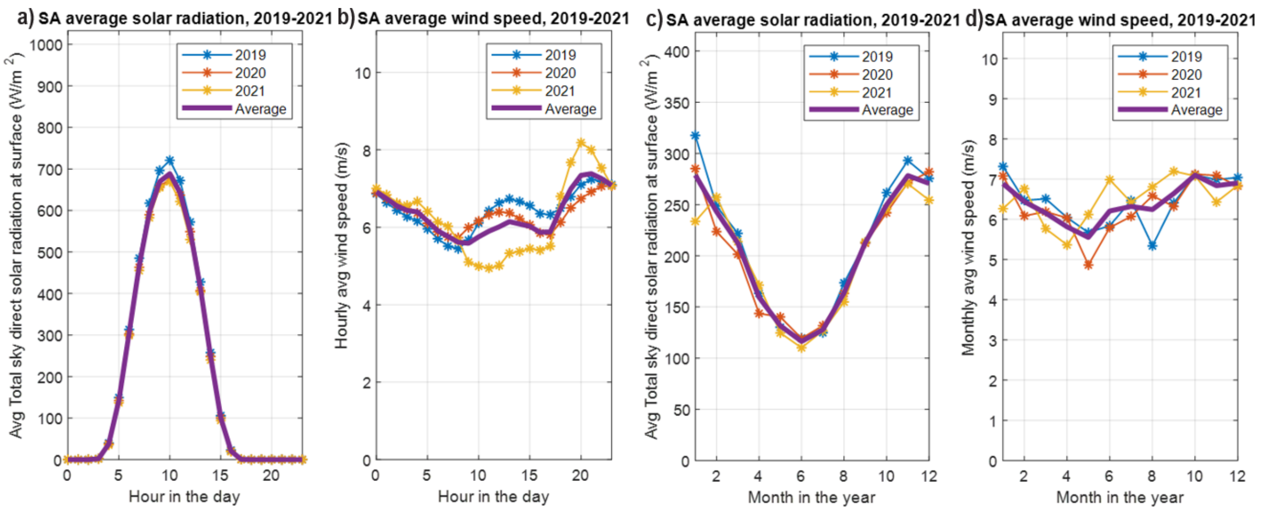

Figure S1: a) average hourly solar radiation, b) average hourly wind speed, c) average monthly solar radiation, d) average monthly wind speed in South Africa.

4. Sensitivity analysis

To understand the influence of energy generation equipment CapEx and design capacity, a sensitivity analysis is performed. Due to the large differences in costs estimates for PV, two scenarios are plotted. These are the base case scenario of 961 USD/kW, and the good case scenario of 618 USD/kWh. LCOE results are plotted for relative battolyser + HB design capacities of 10%, 20%, 30%, and 100%, and varied from a grid of 100% wind / 0% solar, to 0% wind / 100% solar and are illustrated in **figure S2**. Curve smoothing has been applied to the graphs to diminish the noise in the graph, which is the result of several iterative steps in the script. This is however, not performed for the graphs at 10% design capacities, as an incorrect trend would be suggested at low (<5%) percentages of solar in the grid.

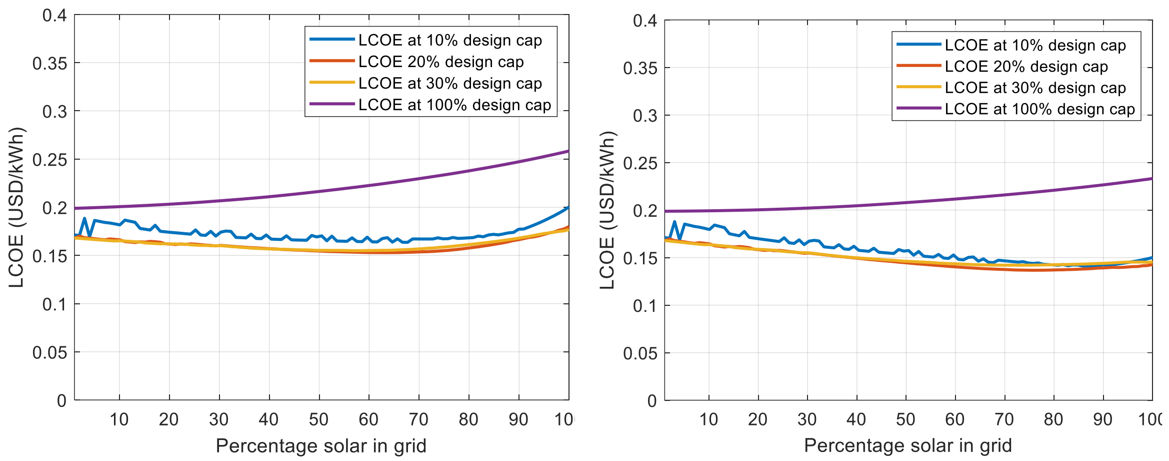

Figure S2: LCOE at relative design capacities of 100%, 30%, 20% and 10% for solar costs of 961 USD/kW (left), and 618 USD/kW (right).

Two interesting phenomena are present in **figure S2**. From **figure S2**, it appears that depending on the costs of solar as well as the design capacity of battolysers + HB equipment, either a full wind configuration, or a hybrid system is the most economically feasible option. It is illustrated that at a good case solar costs scenario, at 20% design capacity of battolysers + HB equipment, an LCOE of 0.15 USD/kWh is reached. The second interesting phenomenon is, an optimum is present by which factor the design capacity of the battolysers and the HB equipment should be undersized. The results suggest that a battolyser and HB capacity of 20-30% of the theoretical maximum is the optimum between battolyser + HB CapEx, and Solar + Wind CapEx. It is expected however, that the optimum is area-dependent, and that results could be different around the world.

Based upon the optimal grid configuration of the base solar costs scenario (30% battolyser design capacity, 37% wind, 63% solar, 961 USD/Kw solar CapEx), a sensitivity analysis is performed to illustrate the effect of the process equipment on the overall LCOE. For this, the process equipment contributing the most to the LCOE are analyzed. The results are illustrated in **Figure S3**. Here, it is presented the percentage of LCOE increase per percent of CapEx and OpEx increase of the respective process equipment.

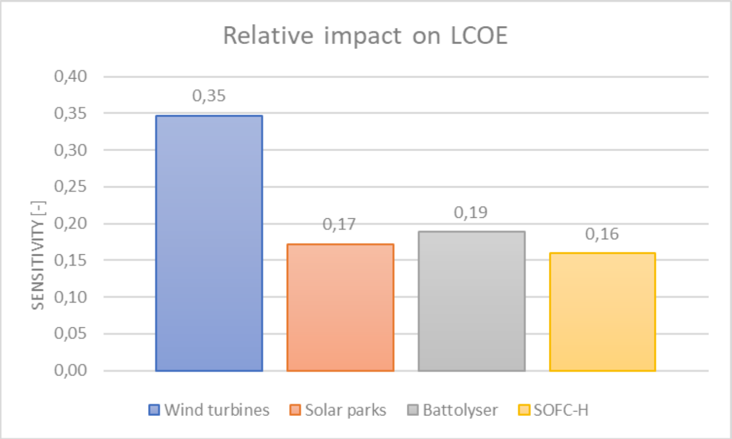

Figure S3: sensitivity analysis of process equipment costs on LCOE.

It becomes clear that although more solar is present in the system as compared to wind energy, the main cost contributor to the LCOE is the wind turbines. This is because the CapEx per peak kW of wind energy is twice as high as compared to solar (1877 USD/kW versus 961 USD/kW), whereas operational costs for wind turbines is almost 4 times higher (2.7% of CapEx/year versus 1.1% of CapEx/year) and additional large maintenance is required for wind turbines after 10 and 15 years costing 17% and 13% of the CapEx, respectively. At last, it is important to state that the costs of wind turbines, solar parks, and battolysers can influence the optimal grid configuration, whereas the costs of the SOFC-H do not impact this optimum. This is because, regardless of the price, the SOFC-H sizing is based upon peak energy demand assuming that no energy is available from wind turbines, PV panels, or the battolyser.

5. References

(1) Sagel, V. N.; Rouwenhorst, K. H. R.; Faria, J. A. Renewable Electricity Generation in Small Island Developing States: The Effect of Importing Ammonia. *Energies (Basel)* **2022**, *15* (9), 3374. https://doi.org/10.3390/en15093374.

(2) Wang, Z.; Deshmukh, A.; Du, Y.; Elimelech, M. Minimal and Zero Liquid Discharge with Reverse Osmosis Using Low-Salt-Rejection Membranes. *Water Res* **2020**, *170*, 115317. https://doi.org/10.1016/j.watres.2019.115317.

(3) Rouwenhorst, K. H. R.; Van der Ham, A. G. J.; Mul, G.; Kersten, S. R. A. Power-to-Ammonia-to-Power (P2A2P) for Local Electricity Storage in 2025. Current Developments, Process Proposal & Future Research Required, University of Twente, 2018. https://doi.org/10.13140/RG.2.2.21200.12800.

(4) Appl, M. Ammonia 1. Introduction. *Ullmann's Encyclopedia of Industrial Chemistry* **2012**, *3*, 1–58. https://doi.org/https://doi.org/10.1002/14356007.a02_143.pub3.

(5) Aziz, M.; Wijayanta, A. T.; Nandiyanto, A. B. D. Ammonia as Effective Hydrogen Storage: A Review on Production, Storage and Utilization. *Energies (Basel)* **2020**, *13* (12), 1–25. https://doi.org/10.3390/en13123062.

(6) Faleh, S.; Khir, · Tahar; Ammar, ·; Brahim, B. Energetic Performance Optimization of a SOFC-GT Hybrid Power Plant. *Arab J Sci Eng* **2017**, *42*, 1505–1515. https://doi.org/10.1007/s13369-016-2363-4.

(7) Rouwenhorst, K. H. R.; Van Der Ham, A. G. J.; Mul, G.; Kersten, S. R. A. Islanded Ammonia Power Systems: Technology Review & Conceptual Process Design. *Renewable and Sustainable Energy Reviews* **2019**, *114*. https://doi.org/10.1016/j.rser.2019.109339.

(8) Ganley, J. C.; Thomas, F. S.; Seebauer, E. G.; Masel, R. I. A Priori Catalytic Activity Correlations: The Difficult Case of Hydrogen Production from Ammonia. *Catal Letters* **2004**, *96* (3–4), 117–122. https://doi.org/10.1023/B:CATL.0000030108.50691.d4.

(9) Sagel, V. N.; Rouwenhorst, K. H. R.; Faria, J. A. Green Ammonia Enables Sustainable Energy Production in Small Island Developing States: A Case Study on the Island of Curaçao. *Renewable and Sustainable Energy Reviews* **2022**, *161*. https://doi.org/10.1016/j.rser.2022.112381.

(10) Zuccari, F.; Orecchini, F.; Santiangeli, A.; Suppa, T.; Ortenzi, F.; Genovese, A.; Pede, G. Well to Wheel Analysis and Comparison between Conventional, Hybrid and Electric Powertrain in Real Conditions of Use. *AIP Conf Proc* **2019**, *2191* (December). https://doi.org/10.1063/1.5138891.

(11) Bañares-Alcántara, R.; Dericks III, G.; Fiaschetti, M.; Grünewald, P.; Lopez, J. M.; Tsang, E.; Yang, A.; Ye, L.; Zhao, S. *Analysis of Islanded Ammonia-Based Energy Storage Systems*; Oxford (United Kingdom), 2015.

(12) Wang, Z.; Deshmukh, A.; Du, Y.; Elimelech, M. Minimal and Zero Liquid Discharge with Reverse Osmosis Using Low-Salt-Rejection Membranes. *Water Res* **2020**, *170*, 115317. https://doi.org/10.1016/j.watres.2019.115317.

(13) *Study on Development of Water Electrolysis in the EU Final Report E4tech Sàrl with Element Energy Ltd for the Fuel Cells and Hydrogen Joint Undertaking*; 2014. https://www.e4tech.com/resources/107-development-of-water-electrolysis-in-the-european-union.php?filter=year%3A2014 (Accessed 20/02/2020)

(14) Schulte-Schulze-Berndt, A.; Krabiell, K. Nitrogen Generation by Pressure Swing Adsorption Based on Carbon Molecular Sieves. *Gas Separation and Purification* **1993**, *7* (4), 253–257. https://doi.org/10.1016/0950-4214(93)80026-S.

(15) Malmali, M.; Le, G.; Hendrickson, J.; Prince, J.; McCormick, A.; Cussler, E. Better Absorbents for Ammonia Separation. *ACS Sustain Chem Eng* **2018**, *6* (5), 6536–6546. https://doi.org/10.1021/acssuschemeng.7b04684.

(16) *COMPARISON OF ELECTRICITY CONSUMPTION FOR AMMONIA AND FREON REFRIGERATION SYSTEMS - CT-Technologies*. https://www.ct-technologies.dk/comparison-of-electricity-consumption-for-ammonia-and-freon-refrigeration-systems/#:~:text=They%20showed%2C%20that%20for%20the,4%2C945%20kWh%20less%20%E2%80%93%2030%25! (Accessed 20/02/2020).

(17) Appl, M. Ammonia 1. Introduction. *Ullmann's Encyclopedia of Industrial Chemistry* **2012**, *3*, 1–58. https://doi.org/https://doi.org/10.1002/14356007.a02_143.pub3.

(18) *Vestas V82-1.65 - 1,65 MW - Wind turbine*. https://en.wind-turbine-models.com/turbines/81-vestas-v82-1.65 (accessed 20/03/2020)

(19) Gomes, J.; Diwan, L.; Bernardo, R.; Karlsson, B. Minimizing the Impact of Shading at Oblique Solar Angles in a Fully Enclosed Asymmetric Concentrating PVT Collector. *Energy Procedia* **2014**, *57*, 2176–2185. https://doi.org/10.1016/J.EGYPRO.2014.10.184.

(20) IEA. *Capital costs of wind in selected emerging economies – Charts – Data & Statistics - IEA*. https://www.iea.org/data-and-statistics/charts/capital-costs-of-wind-in-selected-emerging-economies (accessed 2023-03-23).

(21) Barnard, S. S. S.; Smit, A. M.; Middelberg, S. L.; Botha, M. J. A Cost-Benefit Analysis of Implementing a 54 MW Solar PV Plant for a South African Platinum Mining Company: A Case Study. *Journal of Energy in Southern Africa* **2021**, *32* (3), 76–88. https://doi.org/10.17159/2413-3051/2021/V32I3A11604.

(22) Timilsina, G. R. Demystifying the Costs of Electricity Generation Technologies. **2020**. https://openknowledge.worldbank.org/entities/publication/77e46c80-aea3-5327-b24d-950799c6590c.

(23) Rogers, T.; Ashtine, M.; Koon Koon, R.; Atherley-Ikechi, M. Onshore Wind Energy Potential for Small Island Developing States: Findings and Recommendations from Barbados. *Energy for Sustainable Development* **2019**, *52*, 116–127. https://doi.org/10.1016/j.esd.2019.08.002.
